# Supplementary material for: Resolving Sahelian thunderstorms improves mid-latitude weather forecasts
Source: Nat Commun. 2019 Aug 2;10:3487. doi: 10.1038/s41467-019-11081-4 (PMC6677761; doi:10.1038/s41467-019-11081-4)
Supplement: Supplementary file 1 — Supplementary Information [file 41467_2019_11081_MOESM1_ESM.pdf]

Supplementary Information

Resolving Sahelian thunderstorms improves mid-latitude weather forecasts

Gregor Pante & Peter Knippertz

Supplementary Notes

Supplementary Note 1: Sensitivity of model results on model setup

In order to test whether the changes we discuss in the main paper are mainly due to the differences in resolution or to the use of a convection parameterisation, we performed an additional simulation with the parameterisation switched off at 13 km grid spacing over Africa. Since the grid spacing of nests in ICON has to be a factor of two finer than in the parent domain, we had to nest a 13 km domain over West Africa into a 26 km global domain rather than using 13 km everywhere. Supplementary Figure 2 shows Hovmoeller diagrams of precipitation similar to Fig. 1a–c in the main paper. It clearly demonstrates that 13 km grid spacing without parameterisation (Supplementary Fig. 2b) is able to reproduce single convective systems much more realistically (as compared to the TRMM 3B42 observational data set [1, 2], Supplementary Fig. 2c) than the 26 km (Supplementary Fig. 2a) and the 13 km (Fig. 1a in main paper) global simulations with parameterisation.

For testing the robustness of our results we additionally performed the PARAM and EXPLC simulations for July 2016 with five different model setups as summarised in Supplementary Table 1. The main paper focuses on the combination "v.2.1.00 initDWD". This setup employs the newest model version available when performing the simulations (2.1.00) and an initialisation from operational DWD analysis data. During a three hour time window centred on the actual initialisation time the incremental analysis update (IAU) method is active [3]. The PARAM simulations with v.2.1.00 initDWD emulates the model setup operational in 2016 but benefits from model developments during the past two years. For the reproduction of the operational forecasts for July 2016 we repeat the simulations with older model versions. The operational version was changed on 27 July 2016 from version 2.0.12 to 2.0.14. For the sake of simplicity, the label for this set of simulations is defined as "v.2.0.12" despite the use of two different versions. The configuration remains as described above, however, all namelist switches are adopted to match the operational settings for the two model versions.

The sensitivity of the results to model initialisation is tested by starting simulations from operational ECMWF analysis data of the IFS without the IAU method ("initIFS"). For the initIFS simulations we allow the West African nesting domain in EXPLC to extend to 30°N and omit the European nesting domain in both PARAM and EXPLC. An additional set of initIFS simulations is performed with model version 2.0.15. Except for the initialisation method, all namelist settings conform with the operational configuration for all initIFS simulations as well.

Here we briefly discuss the sensitivity of the model performance to the different setups listed in Supplementary Table 1. Due to the high computational costs, this analysis is restricted to only one month, i.e. July 2016. Therefore it is mostly meant to illustrate general sensitivities but is likely too short for robust statistics. For West Africa, Supplementary Fig. 8a shows a plot similar to Fig. 4c in the main paper (and using the same model version) but restricted to forecast days 1–5. Shadings show DTE as in the main paper and blue dashed lines show the relative difference in specific humidity between EXPLC and PARAM Δq_{rel} (i.e. normalised by absolute values in PARAM to account for the decrease in humidity with height). Consistent with the main paper, this figure shows DTE maxima in the areas of the TEJ, the AEJ and the SHL and a substantial decrease in specific humidity in the mid- and upper-troposphere over the entire domain, exceeding 15% south of 15°N in the middle troposphere. The impact on the AEJ and SHL are almost as strong as for days 1–10 shown in Fig. 4c in the main paper, whereas the major differences in the TEJ representation develop during the second half of the simulations.

Subsequent panels of Supplementary Fig. 8 show differences between the four alternative setups listed in Supplementary Table 1 and the standard setup v.2.1.00 initDWD. Blue lines mark relative difference in specific humidity between

EXPLC and PARAM Δq_{rel} as in Supplementary Fig. 8a to give an orientation for the comparison between the different versions. Shadings indicate differences in DTE, showing whether the impact of switching off the convection scheme in the model gets larger or smaller in the alternative model version. Red lines show the corresponding information for Δq_{rel} or in other words fields are obtained by subtracting the blue dashed lines of each panel with those in panel a. Analysing the DTE and Δq_{rel} fields reveals largest differences between the setups in the area of the TEJ and the SHL. The simulation with an older model version (operational back in 2016) but the same initialisation data shows the overall best agreement with the standard setup (Supplementary Fig. 8b), underlining the importance of initialisation. Sensitivities to convection parameterisation (as indicated by DTE changes) are markedly reduced for the TEJ and moderately for the SHL with slightly higher sensitivities in the upper troposphere. Interestingly, the drying in the upper troposphere is markedly reduced (more than 5%), indicating an important role of the model formulation in this process. Keeping the model version of the standard setup but changing the initialisation data to IFS has a marked impact (Supplementary Fig. 8c). Sensitivities in the TEJ region are still reduced but not as much as in Supplementary Fig. 8b, while sensitivities in the SHL are strongly enhanced. DTE changes are also positive in the mid- and upper-troposphere across large parts of the domain, including a maximum near the core of the AEJ, the strength of which is closely related to the SHL. In the Sahara, tropospheric drying with explicit convection is reduced at low levels but strongly enhanced at mid-levels (more than 15% over the Sahara at about 450 hPa). This is a strong indication that particularly the SHL region is sensitive to initialisation, consistent with the relatively large biases evident for the radiosondes in Tamanrasset (Fig. 2d in main paper, Supplementary Fig. 4–6). It is plausible that longwave radiative effects of water vapour can create relatively large changes in DTE over the dry Sahara in contrast to the much moister southern areas. Now keeping the IFS initialisation data but using the older model version already discussed for Supplementary Fig. 8b, we see a mixture of effects (Supplementary Fig. 8d). The TEJ sensitivity is now even further reduced, while the SHL region shows negative DTE signals in the south and positive signals in the north. Free tropospheric signals in DTE and Δq_{rel} show both upper-tropospheric changes in moisture related to model version as well as mid-tropospheric changes in DTE and moisture related to initialisation. Finally changing the model version to the intermediate v.2.0.15 largely reproduces results for the later v.2.1.00 (compare Supplementary Fig. 8c and e).

To summarise, Supplementary Table 2 shows a qualitative assessment of this sensitivity analysis for the features TEJ, AEJ, SHL and moisture over the Sahara. In addition, a corresponding assessment of the forecast improvement over Europe was made similar to Fig. 3b in the main paper (not shown explicitly here). The summary shows that TEJ sensitivity is reduced for all alternative setups with no clear relationship to the other parameters displayed in Supplementary Table 2. The most striking signal is that all runs initialised with IFS show a large moisture reduction over the mid-level Sahara when switching off the convection parameterisation. This appears to be closely linked to the sensitivity in the SHL region, likely through longwave radiative effects. The v.2.0.12 initIFS experiment shows an additional smaller humidity reduction in the upper troposphere that may compensate for the mid-level radiative effects and thus reduces the SHL sensitivity. The v.2.0.12 initDWD has the same upper-level but no mid-level signal and also shows reduced SHL sensitivity. The latter appears to covary with AEJ signals, likely through the thermal wind relationship. With respect to the performance over Europe, the runs with the highest SHL (and AEJ) sensitivity also show the largest forecast improvement when moving to explicit convection, further underlining the idea that the SHL is an important link to communicate information from Africa to the midlatitudes. The results stress the conclusion that improvements to both atmospheric models and initial conditions (e.g. through better observations or data assimilation over Africa) can potentially contribute to realising better forecast performance in remote regions.

Supplementary Figures

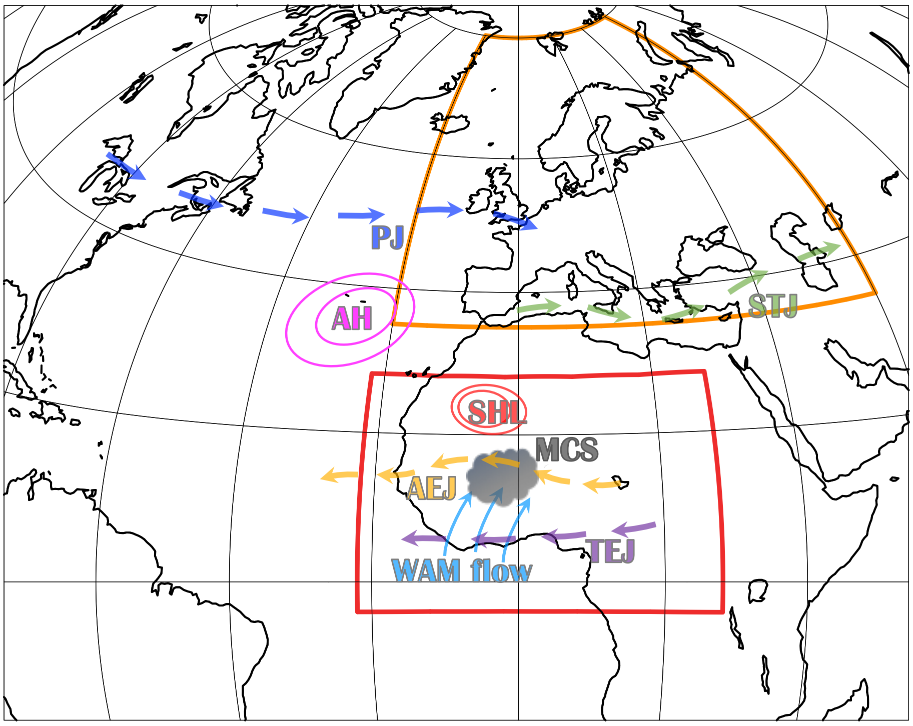

Supplementary Figure 1: Regions and atmospheric features of interest. The two-way nesting domain for EXPLC simulations (4°S–28°N, 22°W–28°E, red box) comprises the entire Sahel region and the Sahara Desert south of the Atlas Mountains. The Darfur Mountains in the eastern part of the domain often trigger mesoscale convective systems (MCSs) that then cross the Sahel westwards [4]. In the south and west the domain reaches out to the Atlantic Ocean. The most important atmospheric features inside the two-way nesting domain are the Saharan heat low (SHL), MCSs, the mid-level African easterly jet (AEJ), the upper-level tropical easterly jet (TEJ) and the low-level West African monsoon (WAM) flow. Changes over Africa can affect extratropical features such as the Azores high (AH), the subtropical jet (STJ) and the polar jet (PJ). The European box for model evaluation (35–80°N, 20°W–60°E) is bordered in orange.

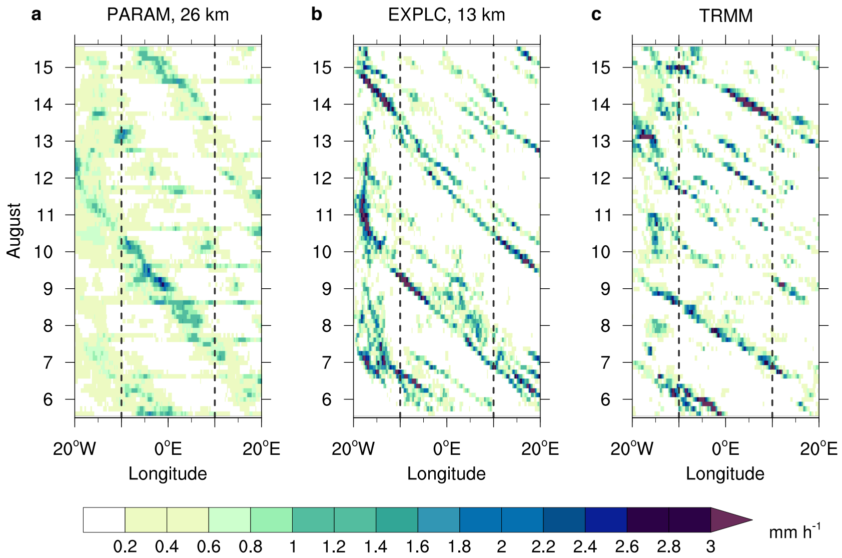

Supplementary Figure 2: Relative importance of resolution vs. convection parameterisation. Hovmoeller diagrams of 3-hourly precipitation as Fig. 1a–c in the main paper for model simulations PARAM but with 26 km grid spacing (**a**) and EXPLC but with 13 km grid spacing (**b**) as well as TRMM 3B42 observations (**c**).

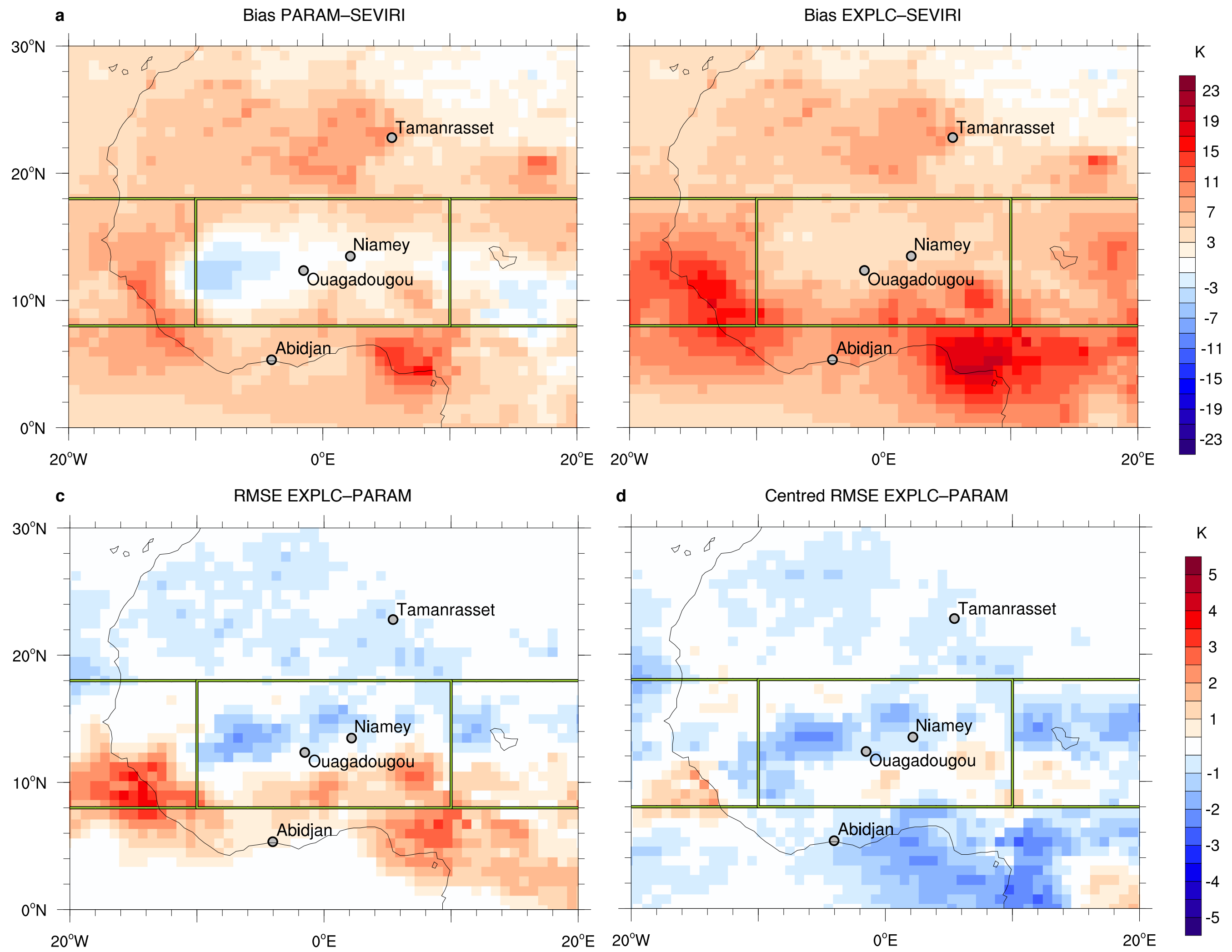

Supplementary Figure 3: Evaluation with satellite-derived brightness temperatures for forecast days 1–10. **a**, Mean bias of PARAM with respect to top-of-atmosphere, infrared (10.8 µm) brightness temperatures from SEVIRI. **b**, Corresponding figure for EXPLC. **c**, Change in mean hourly RMSE between EXPLC and PARAM and **d** corresponding change in CRMSE. Green lines mark the averaging box from Fig. 1d and the latitude band from Fig. 1a–c in the main paper as well as radiosonde station locations used in Fig. 2 in the main paper and Supplementary Fig. 4–6.

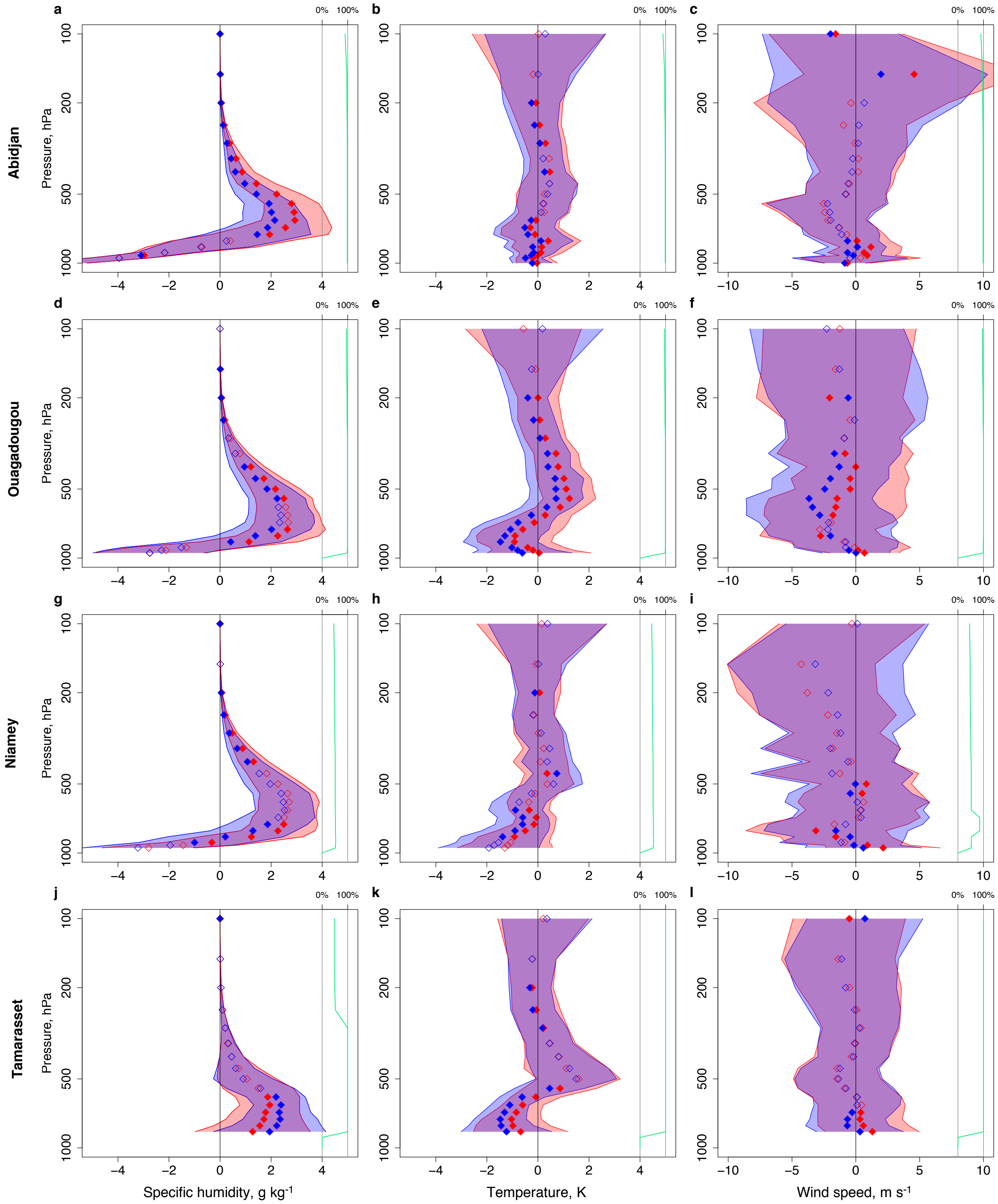

Supplementary Figure 4: Evaluation with radiosondes for the short to medium range. As Fig. 2 in the main paper but for specific humidity (left column), temperature (middle column) and wind speed (right column) and the four stations Abidjan, Ouagadougou, Niamey and Tamanrasset (from top to bottom).

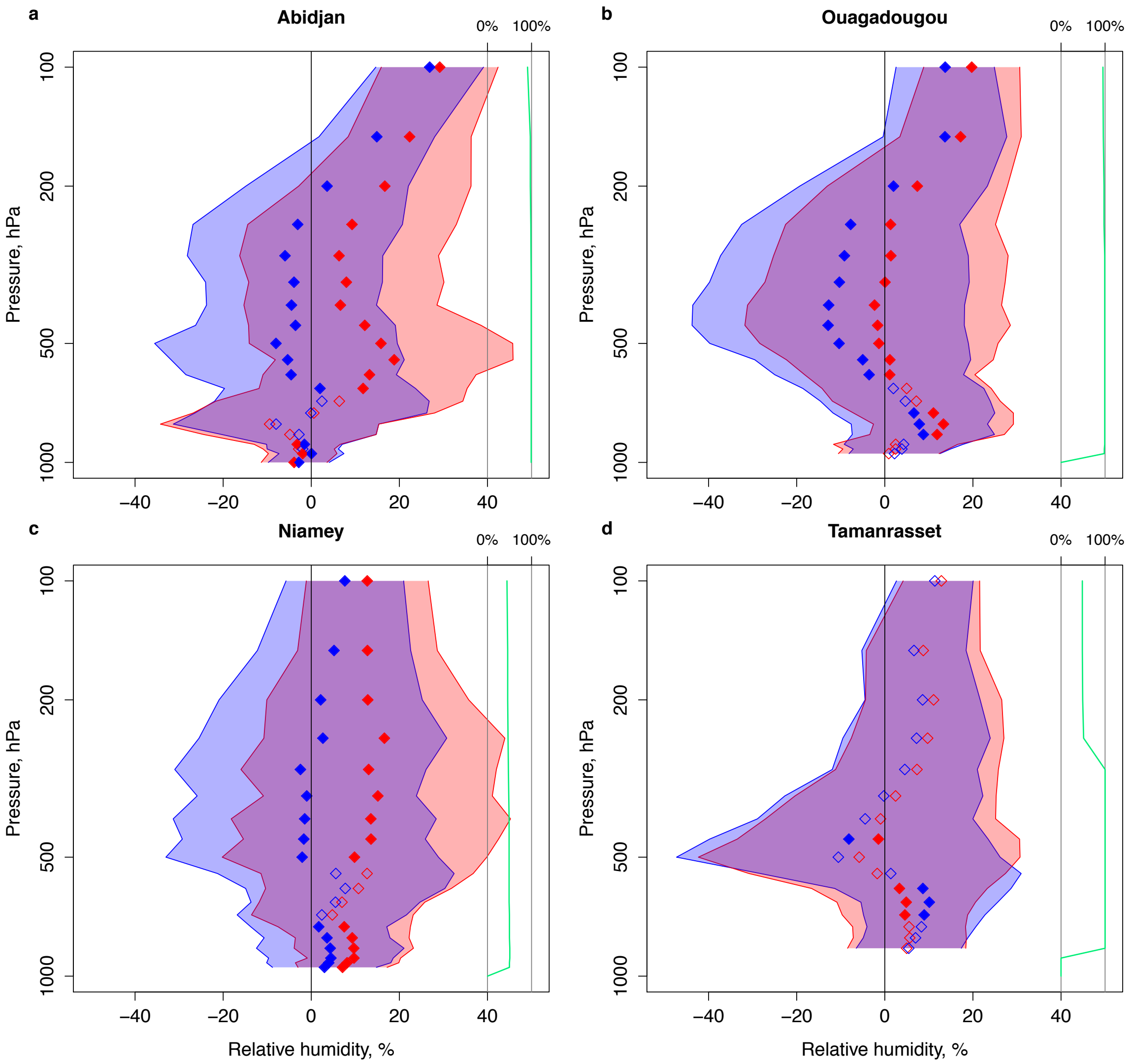

Supplementary Figure 5: Evaluation with radiosondes for the long range. As Fig. 2 in the main paper but for forecast days 6–10.

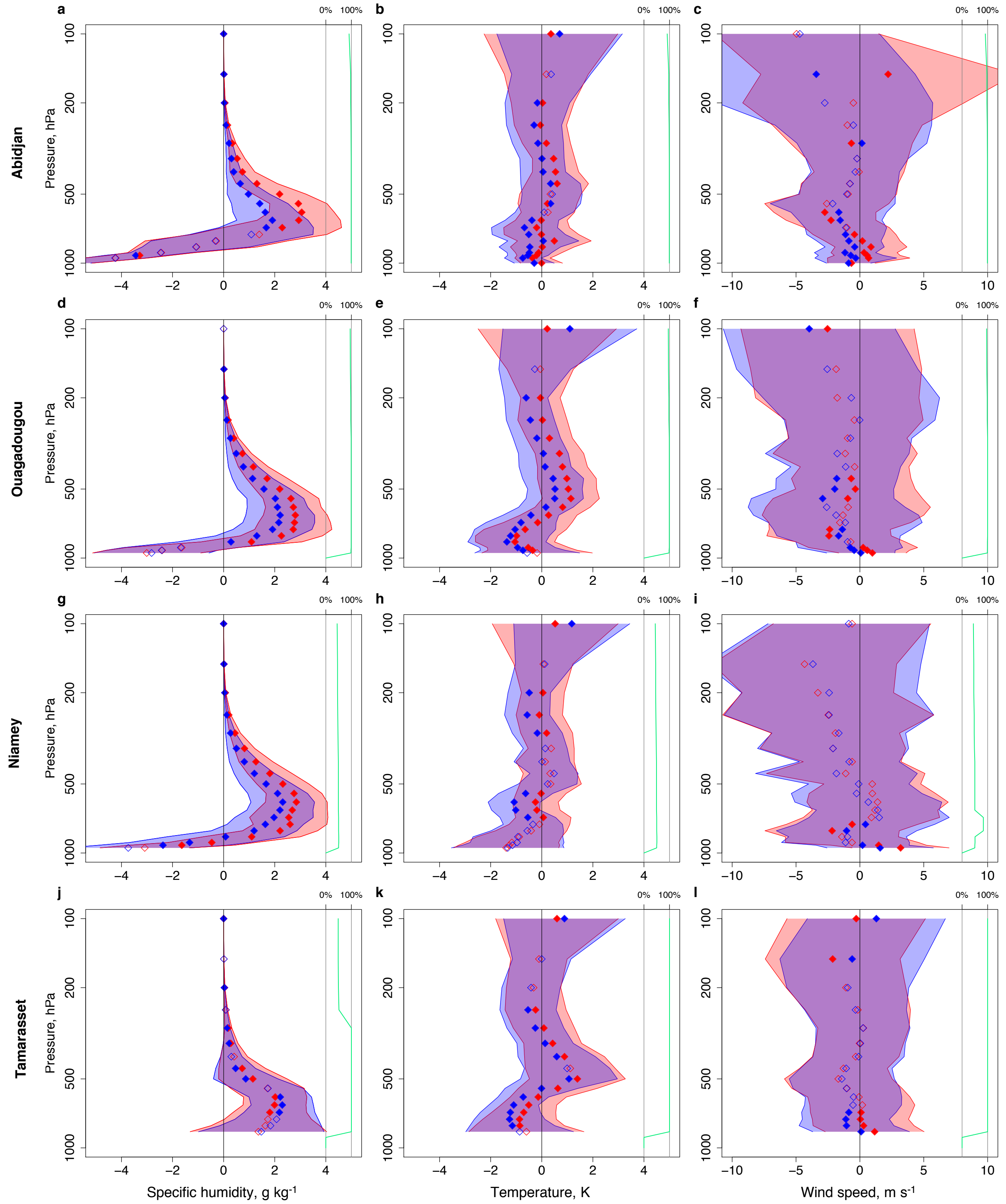

Supplementary Figure 6: Evaluation with radiosondes for the long range. As Supplementary Fig. 4 but for forecast days 6–10.

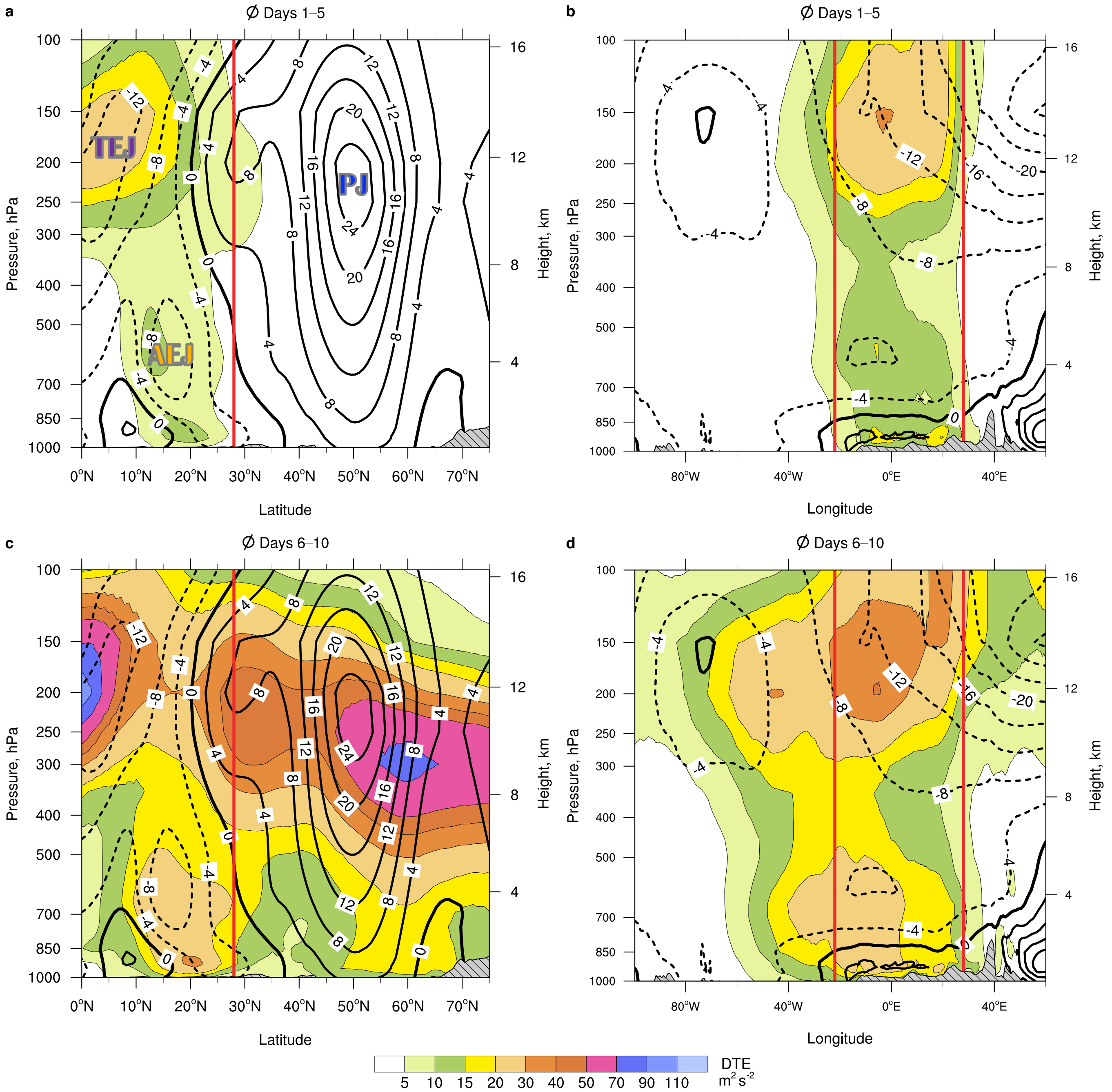

Supplementary Figure 7: Signal propagation along jets. Vertical cross sections of DTE (shading) between EXPLC and PARAM averaged from 40–0°W (left) and from 8–18°N (right) showing the mean over forecast days 1–5 (top) and 6–10 (bottom) and zonal wind speed of PARAM averaged over all 10 forecast days (contour lines labelled in m s^{-1}). Red lines mark the borders of the two-way nesting domain. The positions of AEJ, TEJ and PJ as marked in (a) coincide with the climatological locations of these jets in July, illustrating that the years 2016 and 2017 are likely to be representative for other years.

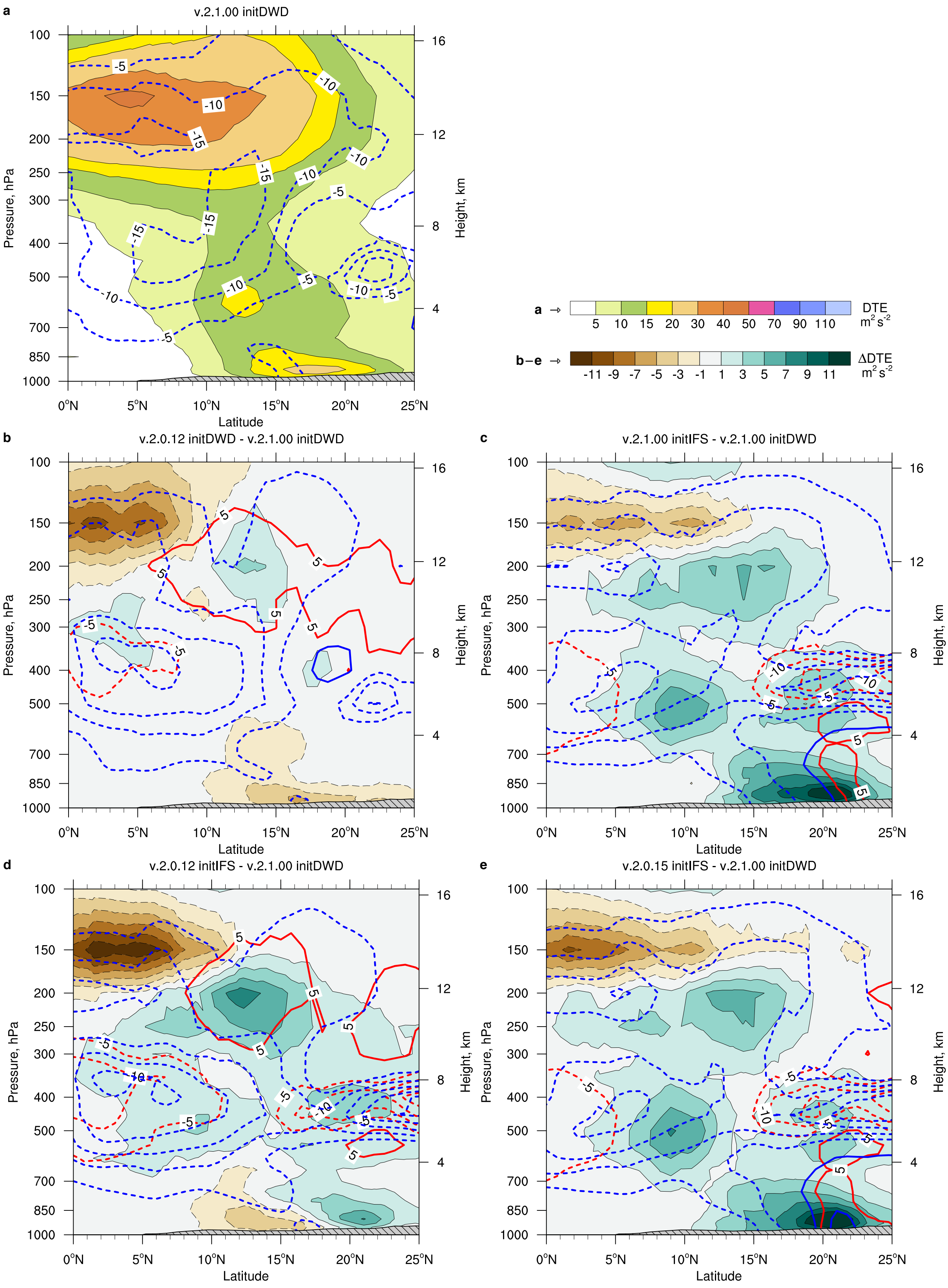

Supplementary Figure 8: Impact of model version and initialisation over West Africa. Vertical cross sections for July 2016 averaged from 10°W–10°E and over forecast days 1–5 with hatched grey shading showing the orography: **a**, DTE (shading) between EXPLC and PARAM and relative difference (i.e. (EXPLC−PARAM)/PARAM) of specific humidity (Δq_{rel}, blue: ±5% intervals) for v.2.1.00 initDWD. **b**, Δq_{rel} (blue: ±5% intervals) for setup v.2.0.12 initDWD (operational in July 2016) and differences in DTE (shading) and Δq_{rel} (red contour lines labelled in %) between setup v.2.0.12 initDWD and the standard setup v.2.1.00 initDWD. Subsequent panels correspond to panel b but show v.2.1.00 initIFS (**c**), v.2.0.12 initIFS (**d**) and v.2.0.15 initIFS (**e**).

Supplementary Tables

Acronym	v.2.1.00 initDWD	v.2.0.12 initDWD	v.2.1.00 initIFS	v.2.0.12 initIFS	v.2.0.15 initIFS
Model version	2.1.00	2.0.12 (1-26 July)<br>2.0.14 (27-31 July)	2.1.00	2.0.12 (1-26 July)<br>2.0.14 (27-31 July)	2.0.15
Initialisation	DWD analysis, IAU	DWD analysis, IAU	IFS analysis	IFS analysis	IFS analysis
Two-way nesting	West Africa and Europe	West Africa and Europe	West Africa	West Africa	West Africa

Supplementary Table 1: ICON model setups. The five different model setups used in this study differ in model version and initialisation method. The IAU is implemented only for initialisation from DWD's own analysis based on the ICON model and not for initialisation from the operational analysis of the IFS. See Supplementary Note 1 for further details.

Model version	v.2.0.12 initDWD	v.2.1.00 initIFS	v.2.0.12 initIFS	v.2.0.15 initIFS
TEJ	−−	−	−−−	−−
SHL	−	++	+	++
Δq_{rel} Sahara, 450 hPa	∘	++	++	++
Δq_{rel} Sahara, 200 hPa	−	∘	−	∘
AEJ	−	+	∘	+
performance over Europe	−−	∘	−	+

Supplementary Table 2: Dependency of the sensitivity to switching off convection parameterisation over Africa on model setup. The first five rows correspond to the signals of DTE and Δq_{rel} shown in Supplementary Fig. 8b–e. The last row is a qualitative assessment of forecast improvement over Europe similar to what is shown in Fig. 3b in the main paper. All signals are relative to the reference setup v.2.1.00 initDWD used for the main paper. Note that higher sensitivities with respect to DTE are reflected in positive values, while higher sensitivities with respect to Δq_{rel} are negative (EXPLC is always drier than PARAM).

Supplementary References

[1] Huffman, G. J. et al. The TRMM multisatellite precipitation analysis (TMPA): quasi-global, multiyear, combined-sensor precipitation estimates at fine scales. *J. Hydrometeorol.* **8,** 38–55 (2007)

[2] Tropical Rainfall Measuring Mission (TRMM), TRMM (TMPA) Rainfall Estimate L3 3 hour 0.25 degree x 0.25 degree V7, Greenbelt, MD, Goddard Earth Sciences Data and Information Services Center (GES DISC), Accessed: 23 May 2018, `http://dx.doi.org/10.5067/TRMM/TMPA/3H/7`.

[3] Reinert, D., Prill, F., Frank, H., Denhard, M. & Zängl, G. *Database reference manual for ICON and ICON-EPS* Version 1.2.2. (Deutscher Wetterdienst, Offenbach am Main, Germany, 2018); `www.dwd.de/DWD/forschung/nwv/fepub/icon_database_main.pdf`

[4] Hodges, K. I. & Thorncroft, C. D. Distribution and statistics of African mesoscale convective weather systems based on the ISCCP Meteosat imagery. *Mon. Weather Rev.* **125,** 2821–2837 (1997)
